# Closed-Loop Identification of Baroreflex Properties in the Frequency Domain

**DOI:** 10.3389/fnins.2021.694512

**Published:** 2021-08-30

**Authors:** Toru Kawada, Keita Saku, Tadayoshi Miyamoto

**Affiliations:** ^1^Department of Cardiovascular Dynamics, National Cerebral and Cardiovascular Center, Osaka, Japan; ^2^Department of Sport and Health Sciences, Faculty of Sport and Health Sciences, Osaka Sangyo University, Osaka, Japan

**Keywords:** baroreflex, white noise, sympathetic nerve activity, arterial pressure, transfer function

## Abstract

The arterial baroreflex system plays a key role in maintaining the homeostasis of arterial pressure (AP). Changes in AP affect autonomic nervous activities through the baroreflex neural arc, whereas changes in the autonomic nervous activities, in turn, alter AP through the baroreflex peripheral arc. This closed-loop negative feedback operation makes it difficult to identify open-loop dynamic characteristics of the neural and peripheral arcs. Regarding sympathetic AP controls, we examined the applicability of a nonparametric frequency-domain closed-loop identification method to the carotid sinus baroreflex system in anesthetized rabbits. This article compares the results of an open-loop analysis applied to open-loop data, an open-loop analysis erroneously applied to closed-loop data, and a closed-loop analysis applied to closed-loop data. To facilitate the understanding of the analytical method, sample data files and sample analytical codes were provided. In the closed-loop identification, properties of the unknown central noise that modulated the sympathetic nerve activity and the unknown peripheral noise that fluctuated AP affected the accuracy of the estimation results. A priori knowledge about the open-loop dynamic characteristics of the arterial baroreflex system may be used to advance the assessment of baroreflex function under closed-loop conditions in the future.

## Introduction

The arterial baroreflex system is one of the most important negative feedback systems that stabilize arterial pressure (AP). Identifying the dynamic characteristics of the arterial baroreflex system is essential to understand the homeostasis of AP in daily activities. When we focus on sympathetic AP controls, the arterial baroreflex system can be divided into two principal subsystems (Ikeda et al., 1996). One is the neural arc subsystem, which defines the relationship between a baroreceptor pressure input and efferent sympathetic nerve activity (SNA). The other is the peripheral arc subsystem, which defines the relationship between SNA and AP. The neural and peripheral arcs can be regarded as the controller and the plant of the arterial baroreflex system, respectively. Under normal physiological conditions, the arterial baroreflex system operates as a closed-loop negative feedback system. Changes in AP affect SNA, whereas changes in SNA, in turn, affect AP. This closed-loop operation hampers the application of a nonparametric frequency-domain system identification method based on a conventional transfer function analysis because the Fourier transformation is mathematically noncausal.

Although we reported a nonparametric frequency-domain closed-loop identification method in previous studies (Kawada et al., 1997; Kawada and Sugimachi, 2016, 2019), the method was described as a set of equations. A certain gap exists between a set of equations and its implementation to programming. This paper, therefore, provides sample codes for the analytical method with sample data files. Although the codes were written in good faith, they could contain unexpected errors. The use may be limited because the codes were developed for analyzing specific datasets. The programs are explained using open-source software GNU Octave and Scilab so that most readers can test the codes on their own (see Appendix). However, the programs were originally developed in commercial software Matlab (MathWorks). The readers are asked to understand those limitations and use the programs on their responsibility. Most figures were drawn on the basis of screenshots so that the readers can easily follow the results of the sample codes.

A dilemma faced frequently in the medical engineering field is that the accuracy of system identification is not parallel with the usefulness of the identification result. Even if the system identification is not perfect, the result may remain useful for diagnosis, risk stratification, prediction of prognosis, and so on. In this article, however, accuracy was sought as a goal of the closed-loop identification. Before explaining the method of closed-loop identification, we will review open-loop dynamic characteristics of the arterial baroreflex system including those in animal models of cardiovascular diseases. The open-loop dynamic characteristics of a system are regarded as the answer to the closed-loop identification. If the closed-loop identification is successful, the identification result will conform to the open-loop dynamic characteristics. After reviewing the methods and results of an open-loop analysis applied to open-loop data, we will discuss a closed-loop analysis. In the section of the closed-loop analysis, we will discuss an open-loop analysis erroneously applied to closed-loop data, followed by a closed-loop analysis applied to closed-loop data.

## Materials and Equipment

The sample data were obtained from past studies (Kawada et al., 1997, 2001). All animal experiments were performed following strict accordance with the Guiding Principles for the Care and Use of Animals in the field of Physiological Sciences, as approved by the Physiological Society of Japan. The experimental protocols were reviewed and approved by the Animal Subject Committee at the National Cerebral and Cardiovascular Center.

The detailed experimental setup was described previously (Kawada et al., 1997, 2001). For the open-loop analysis, the AP was recorded via a catheter-tip, high-fidelity pressure transducer inserted from the femoral artery in anesthetized rabbits. The heart rate (HR) was derived from the AP signal through a cardiotachometer. The carotid sinus baroreceptor regions were isolated from the systemic circulation so that the baroreceptor input pressure was controlled externally with a servo-pump system. The vagal and aortic depressor nerves were sectioned bilaterally at the neck to minimize confounding reflex effects from the aortic arch and cardiopulmonary regions. Efferent SNA was recorded from a branch of the cardiac sympathetic nerve. The nerve signal was amplified and bandpass filtered between 150 to 1,000 Hz. The signal was then full-wave rectified and low-pass filtered with a cut-off frequency of 30 Hz using an analog circuit. Although SNA was expressed in μV, the absolute magnitude of SNA varied depending on the recording conditions, such as the contact between the electrodes and nerve. After the completion of the preparation, the carotid sinus pressure (CSP) was adjusted to AP to obtain the closed-loop operating point of the carotid sinus baroreflex. After that, the CSP was perturbed according to a binary white noise signal around the operating point pressure.

For the closed-loop analysis, the isolated carotid sinuses were connected to the left common carotid artery. Hence, the carotid sinus baroreflex operated as a closed-loop system despite the isolation of the carotid sinus regions. A catheter for blood withdrawal and infusion was inserted from the other femoral artery and placed at the abdominal aorta.

## Open-Loop Analysis

### Time-Series Data

**FIGURE 1:**
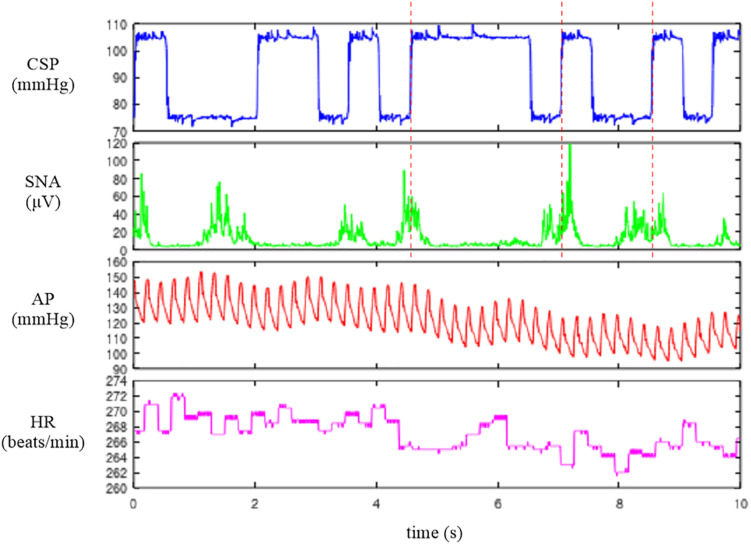
Time series of carotid sinus pressure (CSP), sympathetic nerve activity (SNA), arterial pressure (AP), and heart rate (HR) obtained in one rabbit. CSP was perturbed according to a binary white noise signal. A slight delay was observed between an increment in CSP (vertical dotted lines) and the suppression of the burst activity of SNA.

See the Appendix to set up GNU Octave. Figure 1 shows the first 10 s (2,000 points) of the sample data file “rabbit1-open.dat,” which can be drawn by the following codes:

A = recread(′c:/SampleData\rabbit1-open.dat′, 4);

figure, recplot(A(:, 1:2000), 200);

The user-defined function **recread** reads the data file into a matrix variable A. The first argument specifies the data file name. The folder name needs to be changed according to the location of the sample data file. The second argument specifies the number of channels in the data file as 4. The user-defined function **recplot** plots the time series data. The sampling rate (200 Hz) is specified as a numeric argument. Alternatively, the time-axis data can be provided as the first input argument of **recplot** as follows:

A = recread(′c:/SampleData\rabbit1-open.dat′, 4);

t = (0:2000 − 1) / 200;

figure, recplot(t, A(:, 1:2000));

When neither the sampling rate nor the time-axis data are specified, the abscissa indicates the number of data points. The first channel of Figure 1 indicates CSP, which was changed every 500 ms according to a binary white noise signal. Small ripples were present at high and low values of CSP owing to a limited performance tuning of the servo-pump system. In the second channel, SNA was suppressed during the high CSP level. A closer look indicates a certain delay between the increment of CSP and the suppression of the burst activities of SNA. The third and fourth channels represent the AP and HR signals, respectively. Since the data file is a bare binary file, the number of recorded channels and the format of the stored value need to be known separately. It is advisable to check the waveform of each signal before processing the data. If the data are not decoded correctly (for instance, if the number of recorded channels is wrong), the signals are corrupted.

**FIGURE 2:**
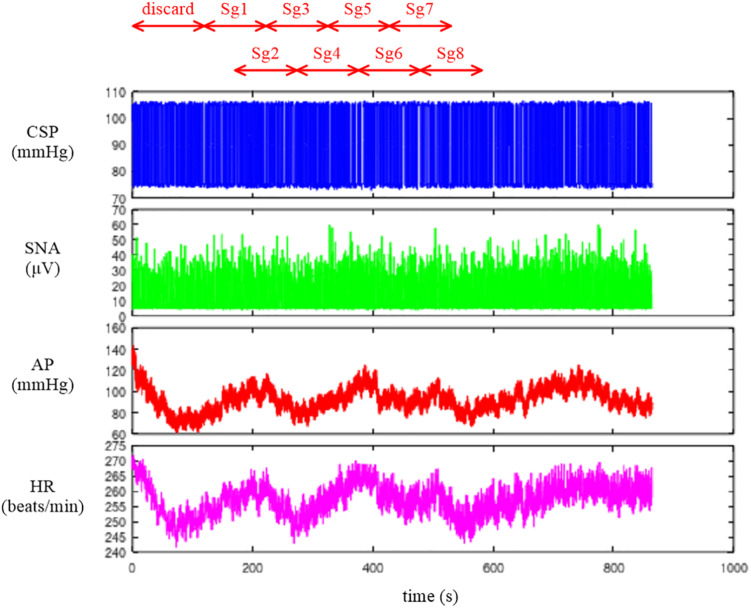
Time series of carotid sinus pressure (CSP), sympathetic nerve activity (SNA), arterial pressure (AP), and heart rate (HR) after 10-Hz resampling. Typically, the data from the first 120 s were discarded to analyze the stationary portion of the data. Sg1 through Sg8 denote the half-overlapping segments for the open-loop transfer function analysis.

Depending on the system under study, the original sampling rate may be unnecessary to capture the overall system dynamic characteristics. Typically, we down-sampled the signals from 200 to 10 Hz. Figure 2 illustrates the whole data of “rabbit1-open.dat” after 10-Hz resampling, which can be obtained from the following codes:

B = recresample(A, 20);

figure, recplot(B, 10)

The user-defined function **recresample** down-samples the data by simply taking an average of each signal every 20 points. After the resampling, the information on the systolic and diastolic pressures of AP is lost. If one wants to analyze the systolic and diastolic pressures, those values need to be obtained before the resampling procedure.

### Conventional Linear Transfer Function Analysis

The neural arc transfer function from CSP to SNA, the peripheral arc transfer function from SNA to AP, and the transfer function of the total reflex arc from CSP to AP were estimated. Although the data before initiating the CSP perturbation were not recorded on the file, a transition from no perturbation to binary perturbation occasionally caused transient changes in the SNA and AP responses. To analyze the stationary portion of the data, we typically discarded the data for the first 120 s (1,200 points) after the initiation of the CSP perturbation. The 10-Hz resampled data were then divided into eight half-overlapping segments of 1,024 points each (Figure 2). The segment length was 102.4 s, which corresponded to the fundamental frequency (*f*_1_) of 0.0098 Hz of the Fourier transformation. Depending on the system under study, the segment length would need to be adjusted. As a rule of thumb, the phase value of the estimated transfer function at the lowest frequency approaches zero radians for a system with a positive response and −π radians for a system with a negative response when the segment length is sufficiently long to capture the system dynamic characteristics. The segment length may need to be prolonged when the phase value at the lowest frequency is not close to either zero or −π radians. An increase in the number of (non-overlapped) segments contributes to reducing the random errors in the transfer function estimation (Bendat and Piersol, 2010). However, too long an observation period would violate the assumption that the system under study can be regarded as time-invariant.

In each segment, a linear trend was removed, and a Hanning window was applied. Next, the frequency spectra of the input signal, *X*(*f*), and the output signal, *Y*(*f*), were obtained through the fast Fourier transformation. Ensemble averages of the input power, *XX*(*f*), output power, *YY*(*f*), and cross spectra between the input and output signals, *YX*(*f*), were calculated over the eight segments. The linear transfer function is then obtained using the following equation (Bendat and Piersol, 2010):

(3.2.1) $$H\left(f\right)=\frac{YX\left(f\right)}{XX\left(f\right)}$$

The magnitude-squared coherence function can also be obtained using the following equation (Bendat and Piersol, 2010):

(3.2.2) $$Coh\left(f\right)=\frac{{\left|YX\left(f\right)\right|}^{2}}{XX\left(f\right)YY\left(f\right)}$$

The coherence function indicates the linear dependence of the output signal on the input signal in the frequency domain. The zero coherence indicates that the output signal is linearly uncorrelated with the input signal. The unity coherence indicates that the output signal is completely explained by the linear dynamics with the input signal.

### Plotting the Transfer Functions

Since the transfer function is complex-valued, it can be described by the modulus (absolute value) and phase angle as follows:

(3.3.1) $$\begin{array}{c} Gain\left(f\right)=abs\left(H\left(f\right)\right)\\ Phase\left(f\right)=angle\left(H\left(f\right)\right) \end{array}$$

The modulus of the transfer function is referred to as gain because it describes the amplitude ratio of the output signal to the input signal at each frequency. The phase of the transfer function describes the difference in the phase between the input and output signals at each frequency.

A caveat in plotting the estimated transfer function is that *f* in the above equations needs to be interpreted as an index of the frequency relative to the fundamental frequency. In addition, the lower bound of the array subscript (the integer number pointing to the element in the array) is 1, not 0, in programming languages that use a matrix as an elementary variable including GNU Octave and Matlab. Hence, the first element of the transfer function, *H*(1), corresponds to the direct current component of the Fourier transformation, which is not used in the transfer function analysis. The second element, *H*(2), represents the transfer function at the fundamental frequency (*f*_1_ = 0.0098 Hz). The (1 + *k*)th element, *H*(1 + *k*), represents the *k*-th harmonic component observed at (*k* × *f*_1_) Hz.

When we use a programming language allowing 0 for the lower bound of the array subscript (such as Microsoft Visual Basic) and implement the Fourier transformation accordingly, the situation is different. The first element becomes *H*(0), and the relation with the frequency index becomes more straightforward; i.e., *H*(*k*) represents the *k*-th harmonic component at (*k* × *f*_1_) Hz.

### Open-Loop Dynamic Characteristics of the Neural and Peripheral Arcs

#### Neural Arc

**FIGURE 3:**
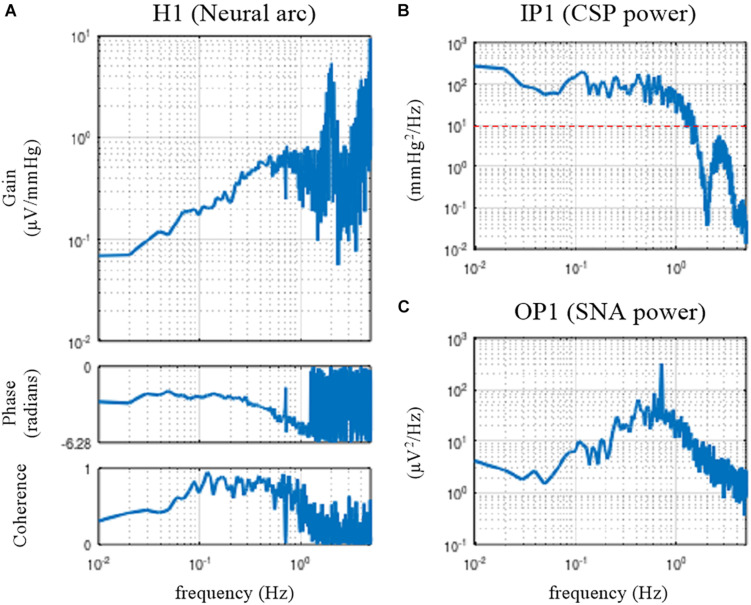
Open-loop transfer function of the baroreflex neural arc, H1 **(A)**, the input power of the carotid sinus pressure (CSP), IP1 **(B)**, and the output power of the sympathetic nerve activity (SNA), OP1 **(C)**. The neural arc revealed high-pass or derivative characteristics, which means that dynamic gain increased as the frequency increased from 0.01 to 0.5 Hz.

Figure 3 illustrates the open-loop dynamic characteristics of the baroreflex neural arc obtained from the following codes:

[H1 C1 IP1 OP1] = calctf(B, [1 2]);

IP1 = IP1 / 10 / 0.3746; OP1 = OP1 / 10 / 0.3746;

figure, subplot(1, 2, 1), tfplot(H1, C1, ′r′);

subplot(2, 2, 2), psplot(IP1), subplot(2, 2, 4), psplot(OP1);

The user-defined function **calctf** calculates the transfer function from channel 1 (row 1, CSP) to channel 2 (row 2, SNA) of the matrix variable B and returns the neural arc transfer function, H1, coherence function, C1, input power spectra, IP1, and output power spectra, OP1. Since the power spectra were calculated on the basis of the unit sampling rate of 1 Hz, they need to be divided by 10 to adjust the values for the 10-Hz resampling data. Further, the power spectra were not corrected for process loss caused by the window function. In the case of the Hanning window, the correction factor of 1/0.3746 may be applied. The denominator of the correction factor for the segment length of 1,024 can be obtained from the following calculation:

c = sum(hanning(1024).^∧^ 2) / 1024

where “^2” denotes the element-wise application of the power of 2.

The user-defined function **tfplot** plots the transfer function and the coherence function. The common logarithm (the logarithm with base 10) of the gain value and the phase value are plotted against the common logarithm of the frequency (a Bode plot). The third input argument of **tfplot** dictates that the phase is plotted in the range from −2π to 0 radians rather than the default range from −π to π radians. The user-defined function **psplot** plots the power spectra. The upper frequency bound of the abscissa displayed by **tfplot** and **psplot** was 5 Hz, which is half of the sampling frequency of 10 Hz. When the sampling rate of the analyzed data is different from 10 Hz, the sampling rate needs to be specified as a numeric argument for **tfplot** and **psplot**. The lower frequency bound of the abscissa displayed by **tfplot** and **psplot** was determined from the length of H1, which equals the segment length of 1,024. By default, the lower frequency bound is the same as the fundamental frequency (10/1,024 = 0.0098 Hz).

The dynamic gain of the neural arc transfer function increased as the frequency increased from 0.01 to 0.5 Hz (Figure 3A). These characteristics are referred to as the high-pass or derivative characteristics of the neural arc (Ikeda et al., 1996). The input power spectra showed that CSP power was fairly constant up to 1 Hz and dropped sharply, making a nadir at 2 Hz (Figure 3B). The nadir occurs at the frequency corresponding to the switching interval of the CSP input signal (500 ms). Since the input power is the denominator to calculate the transfer function (Eq. 3.2.1), the input power at a given frequency needs to be sufficiently large for a stable estimation of the transfer function. A white noise signal, which is rich in frequency components, is an ideal input signal to rigorously test a system within a short observation period (Marmarelis and Marmarelis, 1978). Since a theoretical white noise is unrealizable, a band-limited white noise signal is used in actual applications. In this example, the input power decreased to 10 mmHg^2^/Hz (the red horizontal dotted line) at approximately 1.5 Hz, and hence, the transfer function may be reliable up to 1.5 Hz at the most. The peak in the gain plot at 2 Hz was the artifact due to the division by small numbers at the nadir of the input power. The coherence function reduced to near zero in the frequency range above approximately 1.2 Hz in these data.

The phase of the neural arc transfer function was close to −π radians at the lowest frequency, indicating that the signal inversion for the negative feedback occurred in the neural arc. The inhibitory neurons projecting from the caudal ventrolateral medulla to the rostral ventrolateral medulla are responsible for signal inversions (Masuda et al., 1992). The phase plot is slightly convex-upward relative to −π radians. The phase was delayed as the frequency increased from 0.2 to 1 Hz. A discontinuous phase deflection was observed at approximately 0.7 Hz, which was accompanied by a discontinuous drop in the coherence at the same frequency. The gain plot also shows a small deflection at 0.7 Hz. The SNA power had a sharp peak at 0.7 Hz (Figure 3C). This peak is considered a physiological noise of SNA associated with endogenous respiratory activity, which was linearly uncorrelated with the CSP input signal.

#### Peripheral Arc

**FIGURE 4:**
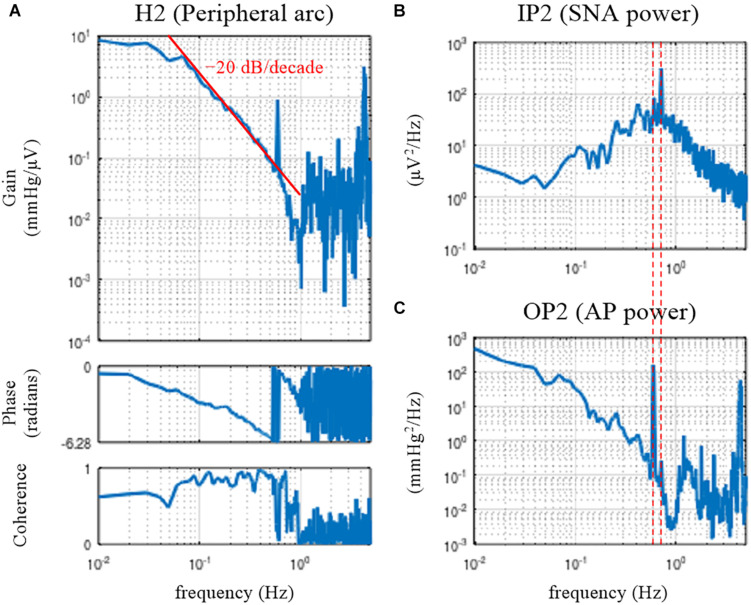
Open-loop transfer function of the baroreflex peripheral arc, H2 **(A)**, the input power of the sympathetic nerve activity (SNA), IP2 **(B)**, and the output power of the arterial pressure (AP), OP2 **(C)**. The peripheral arc revealed low-pass characteristics, with the slope of decreasing gain between 0.1 and 0.5 Hz close to the –20 decibel/decade (the oblique red line). The vertical dotted lines indicate that the frequency of the peak in the SNA power (endogenous respiratory frequency) is not the same as the frequency of the peak in the AP power (artificial ventilation frequency).

Figure 4 illustrates the open-loop dynamic characteristics of the baroreflex peripheral arc obtained from the following codes:

[H2 C2 IP2 OP2] = calctf(B, [2 3]);

IP2 = IP2 / 10 / 0.3746; OP2 = OP2 / 10 / 0.3746;

figure, subplot(1, 2, 1), tfplot(H2, C2, ′r′);

subplot(2, 2, 2), psplot(IP2), subplot(2, 2, 4), psplot(OP2);

The peripheral arc transfer function was calculated between channel 2 (row 2, SNA) and channel 3 (row 3, AP) of the matrix variable B. The dynamic gain of the peripheral arc transfer function decreased as the frequency increased from 0.05 to 1 Hz, indicating the low-pass characteristics of the cardiovascular response to SNA (Figure 4A). A sharp peak was observed at 0.58 Hz in the gain plot. This peak was due to the AP fluctuations at the frequency of artificial ventilation (35 cycles/min). The frequency of the peak in the AP power (0.58 Hz) was different from the frequency of the peak in the SNA power (approximately 0.7 Hz) (Figures 4B,C). The mechanical inflation and deflation of the lungs affected AP independently of SNA. Hence, the peak in the gain plot at 0.58 Hz, which was accompanied by a discontinuous drop of the coherence, does not represent the true characteristics of the AP response to SNA. The decreasing slope of dynamic gain between 0.1 and 0.5 Hz was close to the line of the −20 dB/decade (the oblique red line), which means that the dynamic gain decreased to 1/100 with a 10-fold increase in the frequency.

The phase approached 0 radians at the lowest frequency, suggesting that the steady-state AP response to a step input in SNA was positive. The phase was delayed as the frequency increased and reached −2π radians at approximately 0.6 Hz. Although the phase was wrapped between −2π and 0 radians in this plot, we may unwrap the phase by assuming a continuous phase change along the frequency axis.

### Mathematical Models of Transfer Functions

#### Model Description

Enumerating all gain and phase values on the Bode plot is one way to describe the estimated transfer function. However, by fitting a mathematical model to the estimated transfer function, we can describe the transfer function with only a few parameter values. The neural arc transfer function may be described using the following mathematical model (Kawada et al., 2002):

(3.5.1) $$H1_{model}\left(f\right)=-K_{n}\frac{1+\frac{f}{f_{c1}}j}{{\left(1+\frac{f}{f_{c2}}j\right)}^{2}}e^{-2\pi fL_{n}j}$$

where *j* denotes the unit imaginary number (*j* = $\sqrt{-1}$). *K*_*n*_, *f*_*c*__1_, *f*_*c*__2_, and *L*_*n*_ denote the steady-state gain (in μV/mmHg), the corner frequency relating to the derivative characteristics (in Hz), the corner frequency relating to the high-cut characteristics (in Hz), and the pure delay (in s), respectively. The negative sign in front of *K*_*n*_ indicates that the signal is inverted through the neural arc. The dynamic gain of H1_*model*_ asymptotically approaches *K*_*n*_ as the frequency tends to 0.

The peripheral arc transfer function may be described using a second-order low-pass filter with pure delay as follows (Kawada et al., 2002):

(3.5.2) $$H2_{model}\left(f\right)=\frac{K_{p}}{1+2\zeta \frac{f}{f_{n}}j+{\left(\frac{f}{f_{n}}j\right)}^{2}}e^{-2\pi fL_{p}j}$$

where *K*_*p*_, *f*_*n*_, ζ, and *L*_*p*_ denote the steady-state gain (in mmHg/μV), the natural frequency (in Hz), the damping ratio (dimensionless), and the pure delay (in s), respectively. Depending on the value of ζ, the system behaves as underdamped (0 ≤ ζ < 1), critically damped (ζ = 1), or overdamped (1 < ζ). The dynamic gain of H2_*model*_ asymptotically approaches *K*_*p*_ as the frequency tends to 0.

The structure of the mathematical model is not uniquely determined for a given transfer function. As an example, the denominator of H1_*model*_ could have the same form as that of H2_*model*_, in which case the total number of parameters in H1_*model*_ increases from 4 to 5 (Petiot et al., 2001). Although an increase in the number of parameters of the mathematical model can improve the fitting ability of a model to the estimated transfer function, the meaning of each parameter may become more complicated. Further, possible interdependence between parameters makes the parameter estimation more unstable.

#### Parameter Estimation

The fitting of the mathematical model to the estimated transfer function requires nonlinear least-squares fitting. Although there are many ways to perform such a task, we provided the user-defined function **simplex** based on a method described by Nelder and Mead (1965). Although the convergence of this method is slow, it has a merit in that it does not require a derivative form of the target function. A risk of the convergence to a local minimum needs to be noted, though the risk is not specific to this method.

The error function for the least-squares fitting is difficult to assign because the transfer function is complex-valued. Empirically, we use the following error function, which gives a reasonable fitting result on the Bode plot:

(3.5.3) $$\begin{array}{c} err=\sum_{k=1}^{N}\frac{{\left|\log_{10}\left[H_{est}\left(f\right)\right]-\log_{10}\left[H_{model}\left(f\right)\right]\right|}^{2}}{k}\\ f=f_{1}\times k \end{array}$$

where H_*est*_ and H_*model*_ denote the estimated and model transfer functions, respectively. *N* represents the number of data points from the lowest frequency used for the fitting. During the implementation to the programming, the fact that H_*est*_ is discrete and H_*est*_(1) corresponds to the direct current component needs to be considered.

**FIGURE 5:**
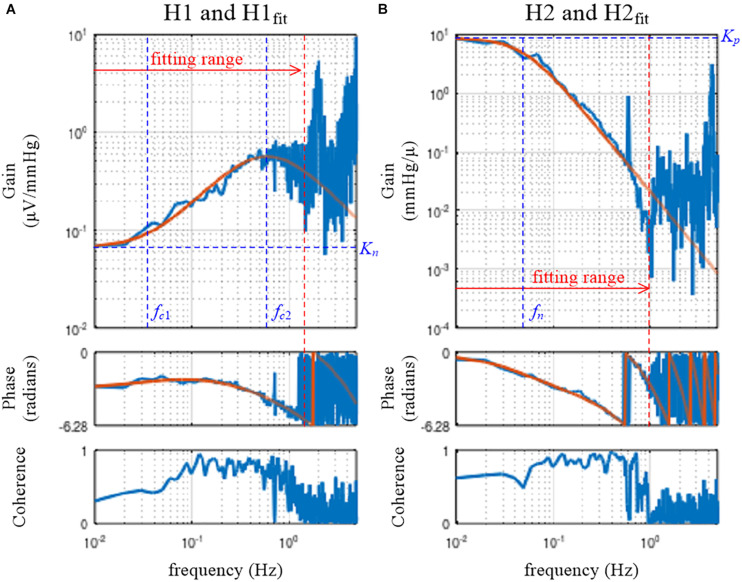
Fitting results of the mathematical models to the estimated transfer functions for the neural arc, H1 **(A)**, and for the peripheral arc, H2 **(B)**. The smooth orange lines represent the transfer functions of the mathematical models, H1_*fit*_ (Eq. 3.5.1) and H2_*fit*_ (Eq. 3.5.2). *K*_*n*_, the steady-state gain of H1_*fit*_; *f*_*c*__1_, the corner frequency describing the derivative characteristics of H1_*fit*_; *f*_*c*__2_, the corner frequency describing the high-cut characteristics of H1_*fit*_; *K*_*p*_, the steady-state gain of H2_*fit*_; *f*_*n*_: the natural frequency of H2_*fit*_.

Figure 5A depicts the fitting result of H1_*model*_ to the neural arc transfer function, which was obtained from the following codes:

x = (0:512)′/(1024/10);

H1model = @(x, p) − p(1) ^∗^ (1+x/p(2) ^∗^ 1j)./(1 + x/p(3) ^∗^ 1j).^∧^2. ^∗^ exp(−2 ^∗^ pi ^∗^ x ^∗^ p(4) ^∗^ 1j);

pout1 = simplex(x(2:151), H1(2:151), [abs(H1(2)) 0.1 1 0.5], H1model);

H1fit = [H1model(x, pout1); zeros(511,1)];

figure, subplot(1, 2, 1), tfplot([H1 H1fit], C1, ′r′);

The first line defines the variable x as the array of frequency values, including 0 for the direct current component. For instance, x(1) is 0, x(2) is the fundamental frequency, x(3) is the second harmonic frequency, and so on. The upper bound of x corresponds to half of the sampling rate; i.e., x(513) = 5.

The second line defines the target function, H1_*model*_, based on Eq 3.5.1. The variable p is the array of parameters, such that p(1), p(2), p(3), and p(4) correspond to *K*_*n*_, *f*_*c*__1_, *f*_*c*__2_, and *L*_*n*_, respectively. In the above definition, H1_*model*_ takes the variables x and p as the input arguments and returns the transfer function values corresponding to x.

The user-defined function **simplex** performs nonlinear least-squares fitting. The first and second arguments of **simplex** are the frequency and the corresponding transfer function values, respectively. Only the first 150 points from the fundamental frequency were used. Note that the subscript range should be “(2:151)”, not “(1:150)”, to skip the direct current component. The third argument of **simplex** gives initial parameter values for *K*_*n*_, *f*_*c*__1_, *f*_*c*__2_, and *L*_*n*_. The initial value for *K*_*n*_ was given as the absolute value of H1(2) rather than a hard number because the dynamic gain of the neural arc varied significantly depending on the absolute amplitude of SNA (e.g., the quality of the SNA recording, position of the electrode relative to the nerve, etc.). Alternatively, we may use arbitrary units for presenting SNA after normalizing SNA values by the average dynamic gain value of H1 in the frequency range, for instance, below 0.03 Hz (Kawada et al., 2001). When the SNA is normalized, the initial value for *K*_*n*_ can be unity. The last argument of **simplex** specifies H1_*model*_ as a target function.

The variable pout1 receives the array of parameter values for *K*_*n*_, *f*_*c*__1_, *f*_*c*__2_, and *L*_*n*_ that attained the minimum value of the error function. In this example, the parameter values of pout1(1) = 0.066, pout1(2) = 0.035, pout1(3) = 0.598, and pout1(4) = 0.198 were obtained.

H1_*fit*_ is the fitting result calculated based on x and pout1. Since the user-defined function **tfplot** determines the frequency axis based on the length of the transfer function data, the length of H1_*fit*_ needs to be adjusted to 1,024 points for its proper presentation on the Bode plot. For this purpose, an array of zeros was added, but this has nothing to do with a zero-padding procedure to increase the frequency resolution. If H1_*fit*_ does not fit to H1 well by visual inspection of the Bode plot, the initial parameter values need to be changed according to the profile of H1. For instance, the initial value for *f*_*c*__2_ may be selected near the frequency of the maximum gain within the fitting range (Figure 5A).

Figure 5B depicts the fitting result of H2_*model*_ to the peripheral arc transfer function, which was obtained from the following codes:

H2model = @(x, p) p(1)./(1+2 ^∗^ p(3) ^∗^ x/p(2) ^∗^ 1j +(x/p(2) ^∗^ 1j).^∧^2). ^∗^ exp(−2 ^∗^ pi ^∗^ x ^∗^ p(4) ^∗^ 1j);

pout2 = simplex(x(2:101), H2(2:101), [abs(H2(2)) 0.1 1.5 1],

H2model);

H2fit = [H2model(x, pout2); zeros(511,1)];

subplot(1, 2, 2), tfplot([H2 H2fit], C2, ′r′);

The target function, H2_*model*_, is defined based on Eq. 3.5.2. For the peripheral arc, only the first 100 points from the fundamental frequency were used to determine the parameters of H2_*model*_. The initial parameter values for *K*_*p*_, *f*_*n*_, ζ, and *L*_*p*_ were given as the third argument of **simplex**. The initial value for ζ was arbitrarily assigned to 1.5, but a different value could be tested. After the fitting was performed, parameter values of pout2(1) = 8.812, pout2(2) = 0.049, pout2(3) = 0.925, and pout2(4) = 0.962 were obtained in this example. Using the variables x and pout2, the fitting result was calculated as H2_*fit*_ and compared with H2 using **tfplot**.

In the user-defined function **simplex**, the fitting weight of the error between the model and estimated transfer functions is reduced by a factor of 1/*k* according to Eq. 3.5.3 because the data points become denser as the frequency increased on the Bode plot. The error function can be modified if needed as an optional input argment to **simplex**:

w = 1./(1:100)′. ^∗^ C2(2:101);

pout2 = simplex(x(2:101), H2(2:101),

[abs(H2(2)) 0.1 1.5 1], H2model, w);

H2fit = [H2model(x, pout2); zeros(511,1)];

subplot(1, 2, 2), tfplot([H2 H2fit], C2, ′r′);

In the above codes, the array for the fitting weight is provided, taking the coherence, C2, into consideration. When the coherence value at a given frequency is low, the fitting weight of the error is reduced. The resulting H2_*fit*_, however, was not changed much in this example.

### Simulation of Baroreflex Dynamic Characteristics

#### Design of a Block Diagram

**FIGURE 6:**
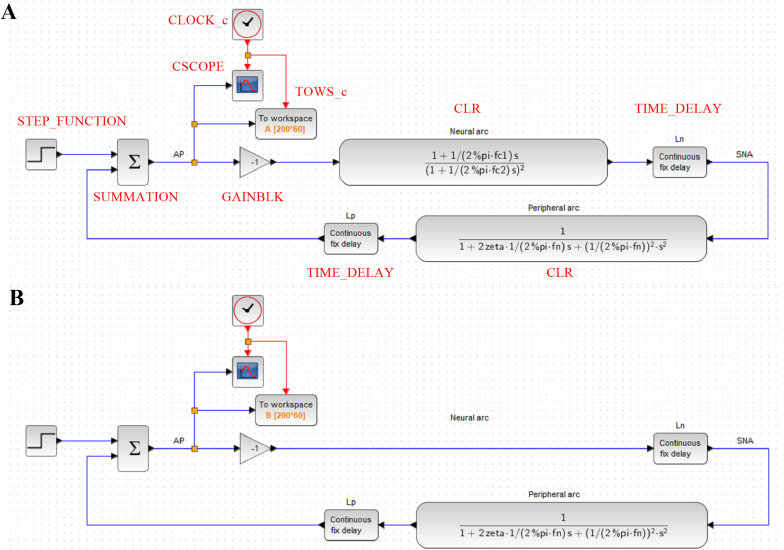
**(A)** A block diagram of the arterial baroreflex system for simulating the closed-loop arterial pressure (AP) change in response to a step pressure perturbation. The red capital letters denote the types of function blocks of Xcos (Scilab). SNA, sympathetic nerve activity; Ln, the pure delay relating to the neural arc; Lp, the pure delay relating to the peripheral arc. **(B)** A block diagram that lacks the dynamic characteristics of the neural arc.

Once the parameters of the model transfer functions are determined, a closed-loop baroreflex response can be simulated by using software such as Xcos (Scilab) and Simulink (Matlab). In the following examples, Xcos was used. Before constructing a block diagram in Xcos, the following variables need to be defined on the Scilab console:

fc1 = 0.035; fc2 = 0.6; Ln = 0.2;

fn = 0.049; zeta = 0.92; Lp = 0.96;

Figure 6A illustrates the block diagram simulating the baroreflex response to a step pressure perturbation (“dynamic_step.zcos”). The neural and peripheral arc transfer functions can be implemented using a function block named CLR. The frequency response of a system described in the ***s***-domain can be obtained by replacing ***s*** with *j*ω, where ω = 2π*f*. For instance, the first-order low-pass filter with a corner frequency of *f*_*c*_ can be converted from the frequency domain to the ***s***-domain as follows:

(3.6.1) $$H\left(f\right)=\frac{1}{1+\frac{f}{f_{c}}j}=\frac{1}{1+\frac{2\pi fj}{2\pi f_{c}}}↔H\left(s\right)=\frac{1}{1+\frac{1}{2\pi f_{c}}s}$$

Hence, the CLR block describing the neural arc, excluding the parameters of dynamic gain and pure delay, can be designed as

(3.6.2) $$H1_{model}\left(s\right)=\frac{1+\frac{1}{2\pi f_{c1}}s}{{\left(1+\frac{1}{2\pi f_{c2}}s\right)}^{2}}$$

Likewise, the CLR block describing the peripheral arc, excluding the parameters of dynamic gain and pure delay, can be designed as

(3.6.3) $$H2_{model}\left(s\right)=\frac{1}{1+2\zeta \frac{1}{2\pi f_{n}}s+{\left(\frac{1}{2\pi f_{n}}\right)}^{2}s^{2}}$$

As a tip of programming, the constant π (3.14159…) is given by “pi” in GNU Octave and Matlab, whereas it is given by “%pi” in Scilab. The pure delay can be implemented using a TIME_DELAY block. For convenience, the delay was defined by a variable *L*_*n*_ or *L*_*p*_ rather than by a hard number. The buffer size of the TIME_DELAY block was increased to 4,096 to avoid an error relating to the short of the buffer size. The gain of the total reflex arc can be assigned using a GAINBLK block. The gain value was set to −1 to reflect the negative feedback nature of the total reflex arc.

The step input can be implemented using a STEP_FUNCTION block. The step time was set at 10, and the final value was set at −30, which means that a pressure disturbance of −30 mmHg was imposed at 10 s. The output from the STEP_FUNCTION block was then combined with the output from the peripheral arc using a SUMMATION block. The number of inputs to the SUMMATION block was set to 2. The output from the SUMMATION block was displayed on a CSCOPE block. The refresh period parameter of the CSCOPE block was set to 60. The CSCOPE block requires a clock input, which was generated by a CLOCK_c block. The interval of the CLOCK_c block was set at 0.005 s (200 Hz), which is the rate at which the visualization is refreshed. The output from the SUMMATION block can also be stored on a workspace variable A using a TOWS_c block. The buffer size of the workspace variable was set to the time resolution (200 Hz) multiplied by the total simulation time (60 s).

#### Role of the Neural Arc

**FIGURE 7:**
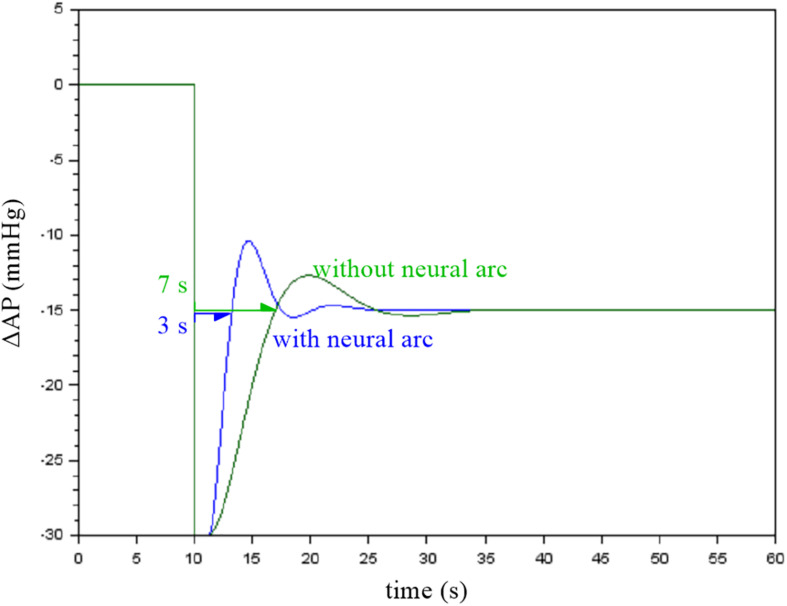
Simulation results showing the effect of the neural arc on the closed-loop arterial pressure (AP) response to a step pressure perturbation. The neural arc shortened the recovery time as assessed by the first point that reached the steady-state response.

The contribution of the neural arc to the baroreflex-mediated dynamic AP response can be examined by removing the CLR block of the neural arc from the simulation (Figure 6B, “dynamic_step_no_neural.zcos”). The simulation result is then stored on another workspace variable B. Figure 7 compares the simulation results with and without the neural arc, which was obtained by the following code on the Scilab console:

t = (0:length(A.values) − 1)′ / 200;

figure, plot(t, [A.values B.values]);

When the first recovery point that exceeded the steady-state response was used to compare the response speed, the removal of the neural arc delayed the recovery of AP by a few seconds in this example. Although the actual baroreflex system is nonlinear and the presence of the pulsatility affects the AP response (Chapleau et al., 1989; Kawada et al., 1992, 2002), the simulation results suggest that the neural arc accelerates the baroreflex-mediated AP response (Ikeda et al., 1996).

#### Static Nonlinearity of the Baroreflex

Although we do not treat the nonlinearity of the baroreflex in this article, the open-loop static characteristics of the neural arc approximated an inverse sigmoidal function. By contrast, the static characteristics of the peripheral arc approximated a straight line within the physiological response range of the baroreflex (Kawada and Sugimachi, 2016). These static characteristics may be implemented in the simulation, which helps interpret certain aspects of the baroreflex characteristics, such as the dependence of the baroreflex gain on the pulsatility of AP (Kawada et al., 1992, 2002) and on AP waveforms (Kawada et al., 2017).

### Open-Loop Dynamic Characteristics of the Total Reflex Arc

#### Total Reflex Arc

The neural and peripheral arc subsystems are serially connected to constitute the total reflex arc. When the two transfer functions, H1 and H2, are serially connected, the overall transfer function, H3, is obtained from the product of H1 and H2 in the frequency domain. In theory, the gain of H3 is a product of gain values of H1 and H2. The phase of H3 is a sum of phase values of H1 and H2. This result can easily be understood using a polar form of the transfer function as follows:

(3.7.1) $$\begin{array}{c} H1\left(f\right)=G1\left(f\right)e^{j\theta 1\left(f\right)}\\ H2\left(f\right)=G2\left(f\right)e^{j\theta 2\left(f\right)}\\ H3\left(f\right)=H1\left(f\right)H2\left(f\right)=G1\left(f\right)G2\left(f\right)e^{j\left[\theta 1\left(f\right)+\theta 2\left(f\right)\right]} \end{array}$$

where G1 and θ1 are the gain and phase values of H1, respectively, and G2 and θ2 are the gain and phase values of H2, respectively.

**FIGURE 8:**
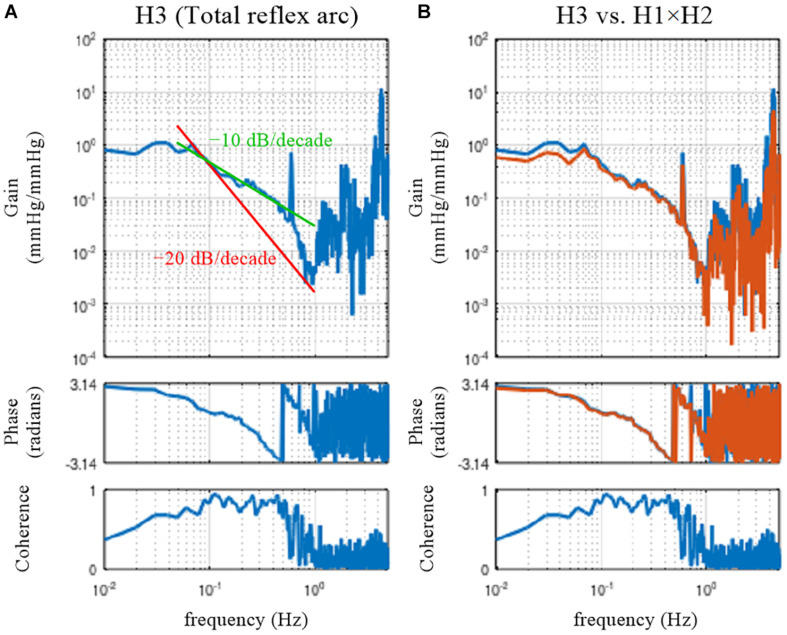
**(A)** Open-loop transfer function of the baroreflex total loop, H3. The slope of decreasing gain between 0.1 and 0.5 Hz was less steep than that of the –20 decibel/decade (the oblique red line) and was close to that of the –10 decibel/decade (the oblique green line). **(B)** Comparison of H3 (the blue lines) and the product of the neural and peripheral arc transfer functions (H1 × H2, the orange lines).

The open-loop dynamic characteristics of the total reflex arc can be directly estimated as a transfer function from CSP to AP using the following codes:

[H3 C3] = calctf(B, [1 3]);

figure, subplot(1, 2, 1), tfplot(H3, C3);

The dynamic gain of the total reflex arc decreased as the frequency increased, indicating the low-pass characteristics (Figure 8A). The peak at 0.58 Hz is an artifact relating to the artificial ventilation frequency. The decreasing slope of dynamic gain between 0.1 and 0.5 Hz was less steep than the line of the −20 dB/decade (the oblique red line) observed for the peripheral arc (Figure 4, left) and close to the line of the −10 dB/decade (the oblique green line). The increasing slope of dynamic gain in the neural arc (Figure 3, left) contributes to increasing dynamic gain values of the total reflex arc between 0.1 and 0.5 Hz. This is the reason why the closed-loop AP response to an exogenous pressure perturbation becomes faster with the neural arc than without (Figure 7; Ikeda et al., 1996).

The phase at the lowest frequency approached π radians, but this result needs to be interpreted as −π radians because of the causality between CSP and AP under baroreflex open-loop conditions. The causality came from the isolated baroreceptor preparation where CSP was controlled independently of AP. The phase delayed as the frequency increased and reached −π radians (or should be interpreted as −3π radians) at approximately 0.5 Hz.

#### Comparison With a Product of Neural and Peripheral Arc Transfer Functions

In actual datases, the total reflex arc transfer function, H3, does not exactly equal the product of H1 and H2 because of the nonlinearity of the system and the presence of noise in the system. The following codes are used to compare H3 and the product of H1 and H2:

[H1 C1] = calctf(B, [1 2]); [H2 C2] = calctf(B, [2 3]); [H3 C3] = calctf(B, [1 3]);

subplot(1, 2, 2), tfplot([H3 H1. ^∗^ H2], C3);

where “H1. ^∗^ H2” indicates the element-wise multiplication of H1 and H2. As can be seen in Figure 8B, there was a deviation of H3 (the blue lines) from the product of H1 and H2 (the orange lines) mainly in the lower frequency range. When the calculations of the cross and power spectra are written down, the three transfer functions are estimated as

(3.7.2) $$\begin{array}{c} H1\left(f\right)=\frac{E\left[SNA\left(f\right)CSP^{*}\left(f\right)\right]}{E\left[CSP\left(f\right)CSP^{*}\left(f\right)\right]}\\ H2\left(f\right)=\frac{E\left[AP\left(f\right)SNA^{*}\left(f\right)\right]}{E\left[SNA\left(f\right)SNA^{*}\left(f\right)\right]}\\ H3\left(f\right)=\frac{E\left[AP\left(f\right)CSP^{*}\left(f\right)\right]}{E\left[CSP\left(f\right)CSP^{*}\left(f\right)\right]} \end{array}$$

where ***E***[] represents the ensemble averaging operation over multiple segments. X^∗^ denotes the complex conjugate of X; i.e., X^∗^ = a − b*j* when X = a + b*j*. Dividing H3 by H1 yields

(3.7.3) $$\frac{H3\left(f\right)}{H1\left(f\right)}=\frac{E\left[AP\left(f\right)CSP^{*}\left(f\right)\right]}{E\left[SNA\left(f\right)CSP^{*}\left(f\right)\right]}$$

**FIGURE 9:**
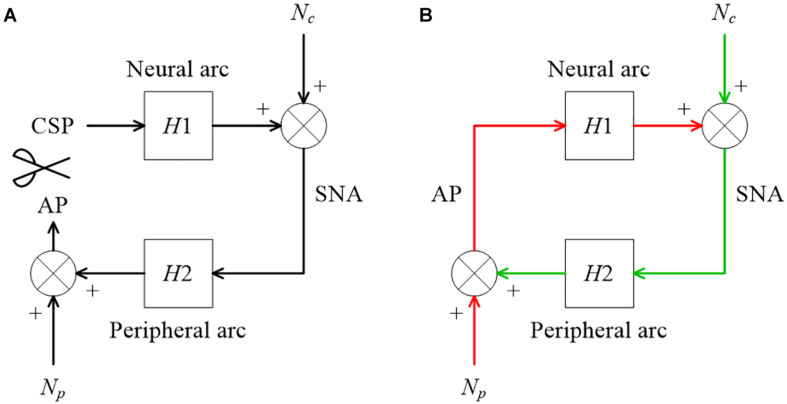
**(A)** A conceptual block diagram describing the open-loop analysis of the carotid sinus baroreflex. Carotid sinus pressure (CSP) was controlled independently of arterial pressure (AP) by using a baroreceptor isolation procedure. H1 and H2 represent the linear transfer function of the neural and peripheral arcs, respectively. SNA, sympathetic nerve activity; N_*c*_, unknown central noise; N_*p*_, unknown peripheral noise. In the open-loop conditions, N_*c*_ does not affect CSP during the estimation of H1, and N_*p*_ does not affect SNA during the estimation of H2. **(B)** A conceptual block diagram describing the closed-loop operation of the carotid sinus baroreflex. A distinct difference from the open-loop experiment (9A) is that N_*c*_ inevitably affects AP through H2 (the green arrows) during the estimation of H1, and N_*p*_ inevitably affects SNA through H1 (the red arrows) during the estimation of H2. These closed-loop signal transductions hamper the application of the conventional open-loop analysis to the closed-loop data.

The right-hand side of Eq. 3.7.3 is not the same as the equation for calculating H2 in Eq. 3.7.2. Figure 9A illustrates a block diagram of a baroreflex open-loop experiment. CSP is controlled independently of AP. N_*c*_ represents the unknown central noise that fluctuates SNA, such as that derived from a central command. N_*p*_ represents the unknown peripheral noise that fluctuates AP, such as that associated with artificial ventilation. If N_*p*_ is absent, and the peripheral arc subsystem is purely linear, the relationship between SNA and AP can be described as

(3.7.4) AP(f)=H2(f)SNA(f)

Substituting AP in Eq. 3.7.3 with Eq 3.7.4 yields

(3.7.5) $$\frac{H3\left(f\right)}{H1\left(f\right)}=\frac{H2\left(f\right)E\left[SNA\left(f\right)CSP^{*}\left(f\right)\right]}{E\left[SNA\left(f\right)CSP^{*}\left(f\right)\right]}=H2\left(f\right)$$

The above transformation assumes that H2 is time-invariant during the observation period and can be treated as a constant with respect to the ensemble averaging operation. Hence, when N_*p*_ is absent and the peripheral arc subsystem is purely linear, H3/H1 mathematically equals H2; i.e., the product of H1 and H2 equals H3.

Next, let us suppose that N_*c*_ is absent and the neural arc subsystem is purely linear. In this case, the relationship between CSP and SNA can be described as

(3.7.6) SNA(f)=H1(f)CSP(f)

Substituting SNA in the calculation of H2 in Eq. 3.7.2 with Eq. 3.7.6 yields

(3.7.7) $$\begin{array}{c} H2\left(f\right)=\frac{E\left[AP\left(f\right){\left(H1\left(f\right)CSP\left(f\right)\right)}^{*}\right]}{E\left[SNA\left(f\right){\left(H1\left(f\right)CSP\left(f\right)\right)}^{*}\right]}\\ =\frac{E\left[AP\left(f\right)CSP^{*}\left(f\right)\right]H1^{*}\left(f\right)}{E\left[SNA\left(f\right)CSP^{*}\left(f\right)\right]H1^{*}\left(f\right)}=\frac{H3\left(f\right)}{H1\left(f\right)} \end{array}$$

Hence, when N_*c*_ is absent and the neural arc subsystem is purely linear, the product of H1 and H2 again equals H3. The fact that H3 was not the same as the product of H1 and H2 in the actual datasets (Figure 8B) suggests that both N_*c*_ and N_*p*_ were present during the experiment. It may be of note that the nonlinear system responses in the neural and peripheral arcs are treated as N_*c*_ and N_*p*_, respectively, from the viewpoint of the linear systems analysis. The nonlinearity of the total reflex arc was estimated in different papers (Moslehpour et al., 2015, 2016a,b).

On a different note, ***E***[] can be replaced with a summation, Σ[], when ***E***[] appears in both the numerator and denominator of the calculation as follows:

(3.7.8) $$\begin{array}{c} XX\left(f\right)=E\left[X\left(f\right)X{\left(f\right)}^{*}\right]=\frac{\sum \left[X\left(f\right)X{\left(f\right)}^{*}\right]}{M}\\ YX\left(f\right)=E\left[Y\left(f\right)X{\left(f\right)}^{*}\right]=\frac{\sum \left[Y\left(f\right)X{\left(f\right)}^{*}\right]}{M}\\ H\left(f\right)=\frac{YX\left(f\right)}{XX\left(f\right)}=\frac{E\left[Y\left(f\right)X{\left(f\right)}^{*}\right]}{E\left[X\left(f\right)X{\left(f\right)}^{*}\right]}=\frac{\sum \left[Y\left(f\right)X{\left(f\right)}^{*}\right]}{\sum \left[X\left(f\right)X{\left(f\right)}^{*}\right]} \end{array}$$

where *X*(*f*) and *Y*(*f*) denote the Fourier transforms of the input and output of the system, respectively, and *M* denotes the number of segments for the ensemble averaging operation.

### Species Differences

**FIGURE 10:**
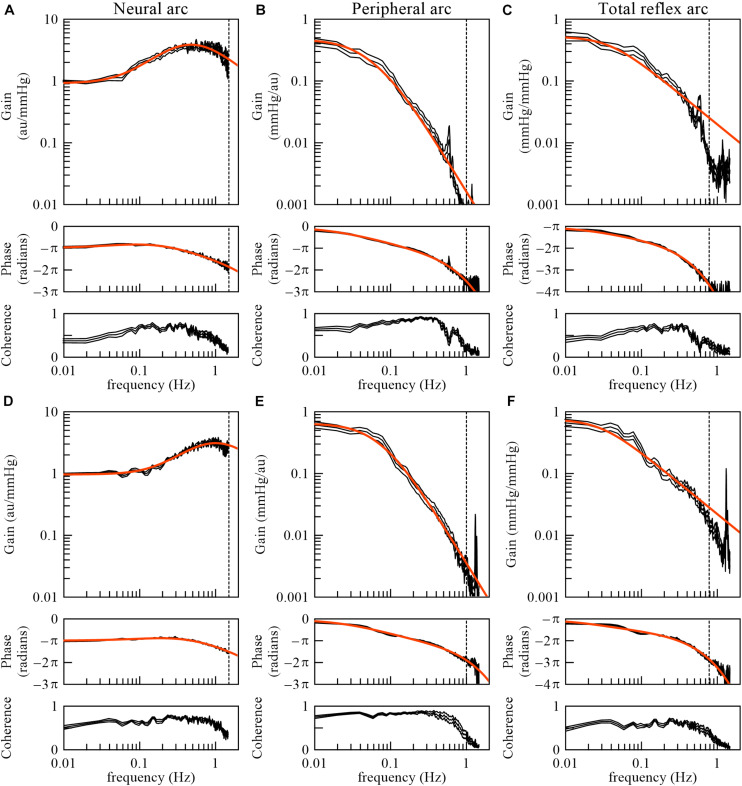
The neural arc **(A)**, peripheral arc **(B)**, and total reflex arc **(C)** transfer functions pooled from 12 Japanese white rabbits. In panels **(B)** and **(C)**, the transfer gain shows a peak at artificial ventilation frequency (35 cycles/min = 0.58 Hz). The neural arc **(D)**, peripheral arc **(E)**, and total reflex arc **(F)** transfer functions pooled from 12 Wistar-Kyoto rats. In panels **(E)** and **(F)**, the transfer gain shows a peak at artificial ventilation frequency (80 cycles/min = 1.33 Hz). The black lines represent mean ± SE values. The smooth orange lines indicate mathematical models fit to the averaged transfer functions. The vertical dotted lines indicate the upper limit of the frequency range used for fitting. The total reflex arc transfer function was modeled with a first-order low-pass filter with pure delay. au, arbitrary units.

The derivative characteristics of the neural arc and the low-pass characteristics of the peripheral arc are commonly observed for rabbits and rats (Kawada and Sugimachi, 2016). Figures 10A–C represent the neural arc, peripheral arc, and total reflex arc transfer functions, respectively, pooled from 12 Japanese white rabbits. In these plots, the SNA values were normalized by the averaged dynamic gain value of the neural arc below 0.03 Hz and expressed in arbitrary units (au). The parameter values of the models (smooth orange lines) fit to the averaged transfer functions were *f*_*c*__1_ = 0.058 Hz, *f*_*c*__2_ = 0.482 Hz, *L*_*n*_ = 0.181 s, *f*_*n*_ = 0.058 Hz, ζ = 1.208, and *L*_*p*_ = 0.782 s. Figures 10D–F represent the neural arc, peripheral arc, and total reflex arc transfer functions, respectively, pooled from 12 Wistar-Kyoto rats. The parameter values were *f*_*c*__1_ = 0.161 Hz, *f*_*c*__2_ = 1.023 Hz, *L*_*n*_ = 0.118 s, *f*_*n*_ = 0.074 Hz, ζ = 1.115, and *L*_*p*_ = 0.474 s. The total reflex arc transfer function may be roughly described with a first-order low-pass filter with pure delay. The corner frequency and the pure delay were 0.038 Hz and 1.410 s, respectively, in rabbits. They were 0.029 Hz and 0.871 s, respectively, in rats. The data derived from Sprague-Dawley rats can be found in a previous article (Kawada and Sugimachi, 2016).

As for the total reflex arc transfer function, dogs also show low-pass characteristics with the natural frequency near 0.02 Hz (Kawada et al., 1992). Changes in vascular properties rather than ventricular properties contribute to the dynamic AP response to CSP (Sakamoto et al., 2015). In humans, the gain of the transfer function from AP to SNA and that from SNA to AP were calculated using closed-loop data (Ando et al., 1997). While the results indicate the derivative characteristics of the neural arc and the low-pass characteristics of the peripheral arc, further research is required regarding the accuracy of those gain estimations, as discussed in section Closed-Loop Analysis.

### Animal Models of Cardiovascular Diseases

Open-loop dynamic characteristics of the carotid sinus baroreflex in animal models of cardiovascular diseases are briefly reviewed. In a rat model of chronic heart failure following myocardial infarction, the baroreflex-mediated dynamic AP regulation was depressed in both the magnitude and response speed, mainly due to the attenuation of the dynamic gain in the peripheral arc (Kawada et al., 2010). The depressed dynamic AP regulation may partly explain why non-compliance with salt and water restriction easily leads to acute decompensation even in stable cardiac patients (Lepage, 2008). Spontaneously hypertensive rats showed well-preserved dynamic characteristics of AP regulation compared with normotensive rats despite having significantly higher baseline AP (Kawada et al., 2011). Hence, changes in the baroreflex dynamic characteristics are not generally predictable from changes in static characteristics of the AP regulation, such as that determined from an inverse sigmoidal relationship between input and output pressures (Sata et al., 2015). In a streptozotocin-induced rat model of type 1 diabetes, *f*_*c*__2_ (the corner frequency for the high-cut characteristics in the neural arc) was lower and ζ (the damping ratio in the peripheral arc) was larger compared with normal rats (Kawada et al., 2018), which suggests derangements in both the neural and peripheral arcs. Depending on types of diseases, functions of the neural and peripheral arcs are differently affected, though we do not have corresponding human data. An assessment of the baroreflex dynamic characteristics in humans will enable the creation of human baroreflex models in health and diseases. The human baroreflex models would add a component of autonomic control to a so-called “digital twin” of a patient, on which we will be able to tailor treatment strategies (Corral-Acero et al., 2020).

## Closed-Loop Analysis

Although the open-loop analysis of the arterial baroreflex is straightforward, it requires baroreceptor isolation preparation and cannot be used in human studies. A closed-loop analysis of the baroreflex dynamic characteristics is a necessary study direction. We examined whether the system dynamic characteristics obtained from closed-loop analysis conformed to those obtained by open-loop analysis.

### Why Does Open-Loop Transfer Function Analysis Not Work Correctly on Closed-Loop Data?

#### Numerical Consideration on Open-Loop Analysis Applied to Closed-Loop Data When Endogenous Noises Exist in Both the Neural and Peripheral Arcs

Figure 9B illustrates a block diagram of the arterial baroreflex under closed-loop conditions. N_*c*_ and N_*p*_ represent the unknown central and peripheral noise, respectively. The relationship between AP and SNA through the neural arc is described in the frequency domain as

(4.1.1) $$SNA\left(f\right)=H1\left(f\right)AP\left(f\right)+N_{c}\left(f\right)$$

The relationship between SNA and AP through the peripheral arc is described as

(4.1.2) $$AP\left(f\right)=H2\left(f\right)SNA\left(f\right)+N_{p}\left(f\right)$$

Why does conventional open-loop transfer function analysis not work correctly on these data? Let us treat AP and SNA as the input and output signals, respectively, to estimate the neural arc transfer function. The ensemble average of the cross spectra between terms of Eq. 4.1.1 and AP yields

(4.1.3) $$E\left[SNA\cdot AP^{*}\right]=H1E\left[AP\cdot AP^{*}\right]+E\left[N_{c}\cdot AP^{*}\right]$$

The description of the frequency is omitted for the sake of brevity. The center dot denotes the multiplication of two complex values (at each frequency) in this article. Since H1 represents the system characteristics of the neural arc and is assumed to be time-invariant during the observation period, it is placed outside the ensemble averaging operation. Under baroreflex closed-loop conditions, ***E***[*N*_*c*_⋅*AP*^∗^] does not diminish asymptotically because N_*c*_ affects AP through the peripheral arc (the green arrows in Figure 9B). Hence, the following equation, which ignores ***E***[*N*_*c*_⋅*AP*^∗^], yields a biased estimation of H1:

(4.1.4) $$H1_{biased}=\frac{E\left[SNA\cdot AP^{*}\right]}{E\left[AP\cdot AP^{*}\right]}$$

The same argument holds for estimating the peripheral arc transfer function under the baroreflex closed-loop conditions. When calculating ensemble averages of the cross spectra between terms of Eq. 4.1.2 and SNA, ***E***[*N*_*p*_⋅*SNA*^∗^] does not disappear because N_*p*_ inevitably affects SNA through the neural arc (the red arrows in Figure 9B). Hence, the following equation, which ignores ***E***[*N*_*p*_⋅*SNA*^∗^], also yields a biased estimation of H2:

(4.1.5) $$H2_{biased}=\frac{E\left[AP\cdot SNA^{*}\right]}{E\left[SNA\cdot SNA^{*}\right]}$$

#### Numerical Consideration on Open-Loop Analysis Applied to Closed-Loop Data When Endogenous Noise Exists Only in the Neural or Peripheral Arc

When N_*p*_ is absent and N_*c*_ alone activates the baroreflex system, the input-output relationship through the peripheral arc becomes

(4.1.6) AP(f)=H2(f)SNA(f)

Calculating ensemble averages of the cross spectra between terms of Eq. 4.1.6 and SNA yields

(4.1.7) $$E\left[AP\cdot SNA^{*}\right]=H2E\left[SNA\cdot SNA^{*}\right]$$

Since N_*p*_ = 0, the estimation of H2 using the right-hand side of Eq. 4.1.5 is not biased in this case:

(4.1.8) $$H2=\frac{E\left[AP\cdot SNA^{*}\right]}{E\left[SNA\cdot SNA^{*}\right]}$$

Next, calculating ensemble averages of the cross spectra between terms of Eq. 4.1.6 and AP yields

(4.1.9) $$E\left[AP\cdot AP^{*}\right]=H2E\left[SNA\cdot AP^{*}\right]$$

Comparing Eq. 4.1.9 with Eq. 4.1.4, we have

(4.1.10) $$H1_{biased}=\frac{E\left[SNA\cdot AP^{*}\right]}{E\left[AP\cdot AP^{*}\right]}=\frac{1}{H2}$$

Hence, H1_*biased*_ is an inverse of H2 and does not reflect H1 at all. To summarize, when only N_*c*_ exists, H2 can be estimated accurately, but H1_*biased*_ simply returns an inverse of H2 regardless of the profile of H1. Conversely, when only N_*p*_ exists, H1 can be estimated accurately, but H2_*biased*_ returns an inverse of H1. In the actual datasets, the relative accuracy of H1_*biased*_ and H2_*biased*_ depends on the relative magnitude of N_*c*_ and N_*p*_ (Kamiya et al., 2011).

#### Erroneous Application of Open-Loop Analysis to Closed-Loop Data

**FIGURE 11:**
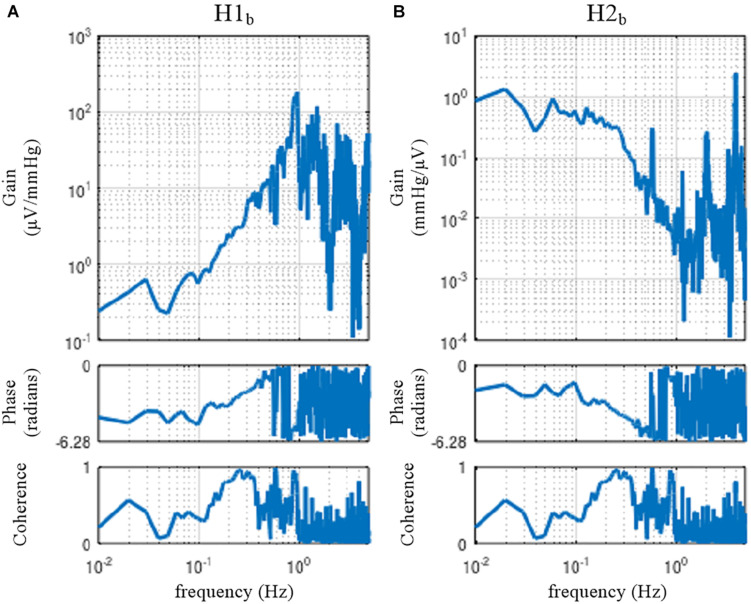
The neural arc **(A)** and peripheral arc **(B)** transfer functions as estimated from an erroneous application of conventional open-loop analysis to the closed-loop data obtained from an anesthetized rabbit. H1_*b*_ and H2_*b*_ were derived from Eqs. 4.1.4 and 4.1.5, respectively. A fixed relation of angle(H1_*b*_) = –angle(H2_*b*_) was observed. C1_*b*_ equals C2_*b*_.

The sample data file “rabbit2-vx.dat” contains SNA and AP signals obtained from an anesthetized rabbit after vagotomy but before isolating the carotid sinus baroreceptor regions. Hence, the carotid sinus baroreflex operated as a closed-loop feedback system. Figure 11 illustrates the results of an erroneous application of the conventional open-loop analysis to the closed-loop data, which was derived from the following codes:

A = recread(′c:/SampleData/rabbit2-vx.dat′, 4);

B = recresample(A, 20);

[H1b C1b] = calctf(B, [3 2]); [H2b C2b] = calctf(B, [2 3]);

figure, subplot(1, 2, 1), tfplot(H1b, C1b, ′r′); subplot(1, 2, 2), tfplot(H2b, C2b, ′r′);

In the gain plots, H1_*b*_ and H2_*b*_ roughly captured the derivative characteristics of the neural arc and the low-pass characteristics of the peripheral arc, respectively. However, the phase plot of H1_*b*_ was convex downward rather than upward in the frequency range from 0.01 to 0.5 Hz. Numerically, the phase of the transfer function is derived from the cross spectra in the numerator of the transfer function calculation because the input power spectra in the denominator are real-valued. For the application of the conventional open-loop transfer function analysis to the closed-loop data, the phase of H1_*b*_ was derived from ***E***[*SNA*⋅*AP*^∗^] (Eq. 4.1.4), whereas the phase of H2_*b*_ was derived from ***E***[*AP*⋅*SNA*^∗^] (Eq. 4.1.5), which is (***E***[*SNA*⋅*AP*^∗^])^∗^. Because angle(X) = −angle(X^∗^), there is a fixed relation of angle(H1_*b*_) = −angle(H2_*b*_). Hence, the phase plots in Figure 11 cannot be simultaneously correct for H1_*b*_ and H2_*b*_ over the entire frequency range. This means that gain plots cannot be simultaneously correct for H1_*b*_ and H2_*b*_, either, because gain and phase are inseparable quantity of a transfer function. We cannot tell about the causality between SNA and AP from the results of the conventional open-loop analysis applied to the closed-loop data. As a numerical consequence (Eq. 3.2.2), C1_*b*_ equals C2_*b*_.

**FIGURE 12:**
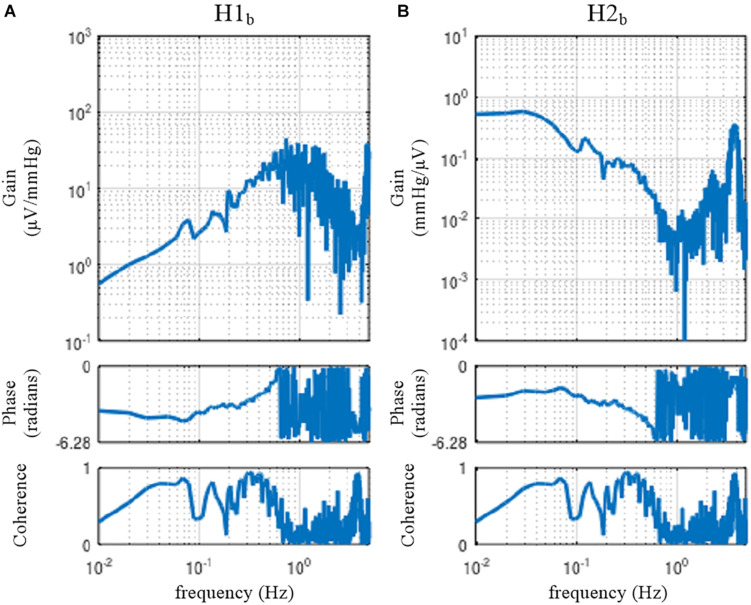
The neural arc **(A)** and peripheral arc **(B)** transfer functions estimated by an erroneous application of conventional open-loop analysis to the closed-loop data obtained from a conscious rabbit. H1_*b*_ and H2_*b*_ were derived from Eqs. 4.1.4 and 4.1.5, respectively. There was a fixed relation of angle(H1_*b*_) = –angle(H2_*b*_). C1_*b*_ equals C2_*b*_.

When the rabbit is under conscious conditions, the magnitudes of N_*c*_ and N_*p*_ and their balance may be different from those under anesthetized conditions. The sample data file “rabbit3-awake.dat” contains SNA and AP signals obtained from a conscious rabbit sitting in a small box. The SNA was recorded from a branch of the renal nerve via a pair of stainless-steel wire electrodes implanted using a sterile procedure a few days before. The AP was measured via a catheter inserted into the central ear artery under local anesthesia. The carotid sinus baroreceptor regions and the vagal and aortic depressor nerves were kept intact. The result of an erroneous application of the conventional open-loop analysis to the closed-loop data is shown in Figure 12. Although we expected that the situations of the identifiability of the system dynamic characteristics would differ between conscious and anesthetized conditions, Figure 12 seems qualitatively similar to Figure 11, despite the differences in the experimental preparations (intact vs. sectioned vagi, the recording of renal vs. cardiac sympathetic nerve).

### Closed-Loop Identification With Exogenous Pressure Perturbation

#### Numerical Consideration

**FIGURE 13:**
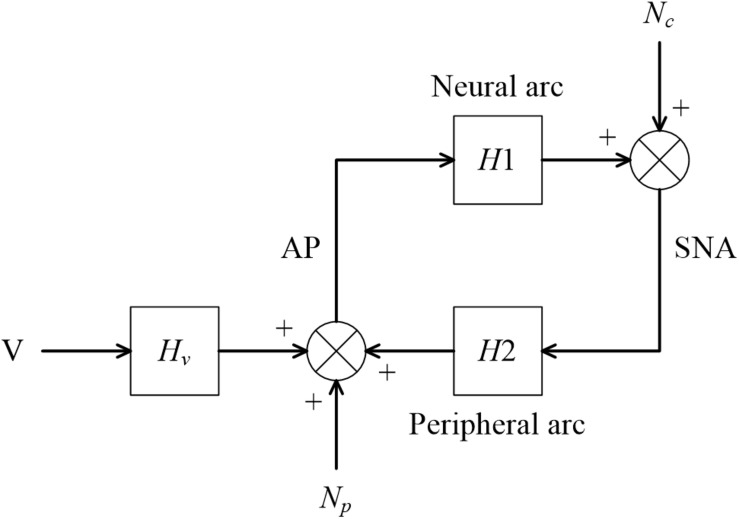
A conceptual block diagram describing the closed-loop identification of the carotid sinus baroreflex using an exogenous pressure perturbation. H1 and H2 represent the linear transfer function of the neural and peripheral arcs, respectively. V and H_*v*_ represent the command for the exogenous pressure perturbation and the transfer function from the command signal to arterial pressure (AP), respectively. N_*c*_, unknown central noise; N_*p*_, unknown peripheral noise; SNA, sympathetic nerve activity.

Figure 13 illustrates a block diagram of the arterial baroreflex under closed-loop conditions with an exogenous pressure perturbation. V and H_*v*_ denote the command of the exogenous pressure perturbation and the transfer function from V to AP, respectively. The relationship between SNA and AP through the peripheral arc is described in the frequency domain as

(4.2.1) $$AP\left(f\right)=H2\left(f\right)SNA\left(f\right)+N_{p}\left(f\right)+H_{\nu }\left(f\right)V\left(f\right)$$

For the closed-loop identification, calculating ensemble averages of the cross spectra between terms of Eq. 4.1.1 and V yields

(4.2.2) $$E\left[SNA\cdot V^{*}\right]=H1E\left[AP\cdot V^{*}\right]+E\left[N_{c}\cdot V^{*}\right]$$

When V is a white noise signal, N_*c*_ and V become statistically independent, and ***E***[*N*_*c*_⋅*V*^∗^] asymptotically diminishes. Hence, the neural arc transfer function can be estimated from the following equation:

(4.2.3) $$H1=\frac{E\left[SNA\cdot V^{*}\right]}{E\left[AP\cdot V^{*}\right]}$$

Once H1 is estimated, N_*c*_ can be estimated from Eq. 4.1.1 as

(4.2.4) $$N_{c}=SNA-H1\cdot AP$$

Calculating ensemble averages of the cross spectra between terms of Eq. 4.2.1 and N_*c*_ yields

(4.2.5) $$E\left[AP\cdot N_{c}^{*}\right]=H2E\left[SNA\cdot N_{c}^{*}\right]+E\left[N_{p}\cdot N_{c}^{*}\right]+H_{\nu }E\left[V\cdot N_{c}^{*}\right]$$

In the above equation, ***E***[*N*_*p*_⋅*N*_*c*_^∗^] asymptotically diminishes because all linear couplings between SNA and AP are expressed by H1 and H2 in the diagram shown in Figure 13, and N_*p*_ and N_*c*_ are linearly uncorrelated, by definition. The last term, ***E***[*V*⋅*N*_*c*_^∗^], also asymptotically diminishes when V is a white noise signal. Accordingly, the peripheral arc transfer function can be estimated from the following equation:

(4.2.6) $$H2=\frac{E\left[AP\cdot N_{c}^{*}\right]}{E\left[SNA\cdot N_{c}^{*}\right]}$$

It should be noted that the reliability of the H2 estimation depends on the properties of N_*c*_. Inputs from higher brain centers generate N_*c*_, as evidenced by the variation of SNA under a fixed CSP (Moslehpour et al., 2016b). However, if there is not enough power of N_*c*_ in a certain frequency range, the H2 estimation becomes unreliable because of divisions by small numbers. For calculating H2, a direct estimation of N_*c*_ is not necessary. Substituting N_*c*_ in Eq. 4.2.6 with Eq. 4.2.4 yields

(4.2.7) $$\begin{array}{c} H2=\frac{E\left[AP\cdot {\left(SNA-H1\cdot AP\right)}^{*}\right]}{E\left[SNA\cdot {\left(SNA-H1\cdot AP\right)}^{*}\right]}\\ =\frac{E\left[AP\cdot SNA^{*}\right]-H1^{*}E\left[AP\cdot AP^{*}\right]}{E\left[SNA\cdot SNA^{*}\right]-H1^{*}{\left(E\left[AP\cdot SNA^{*}\right]\right)}^{*}} \end{array}$$

#### Application of Closed-Loop Identification

**FIGURE 14:**
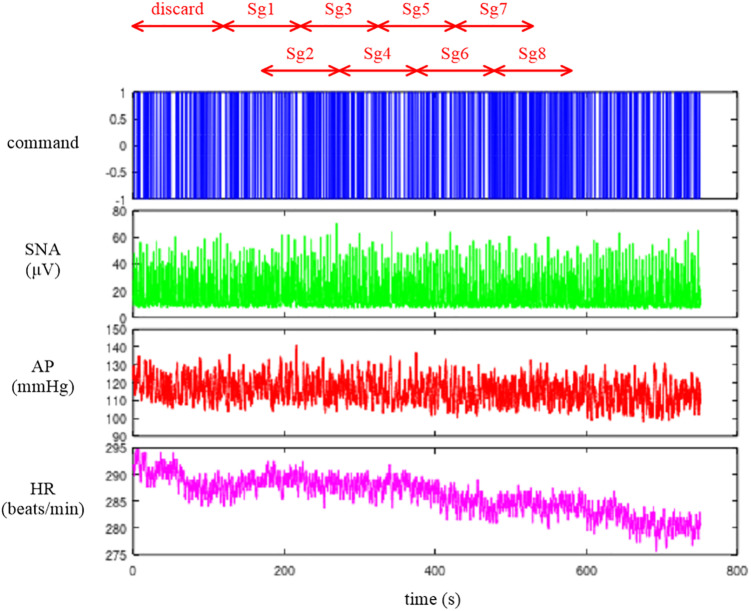
Time series of a command signal for an exogenous pressure perturbation, sympathetic nerve activity (SNA), arterial pressure (AP), and heart rate (HR) after 10-Hz resampling. The data from the first 120 s were discarded to analyze the stationary portion of the data. Sg1 through Sg8 denote the half-overlapping segments for the closed-loop transfer function analysis.

The sample data file “rabbit4-closed.dat” contains SNA and AP signals during an exogenous pressure perturbation induced by blood withdrawal and infusion obtained in an anesthetized and vagotomized rabbit. Figure 14 depicts the 10-Hz resampled data of the whole recording. The first channel represents the command signal for blood infusion (a positive value) and withdrawal (a negative value). The command was changed according to a binary white noise signal with a switching interval of 1 s. The second and third channels represent SNA and AP signals, respectively. In the fourth channel, HR showed a decreasing trend in this example.

**FIGURE 15:**
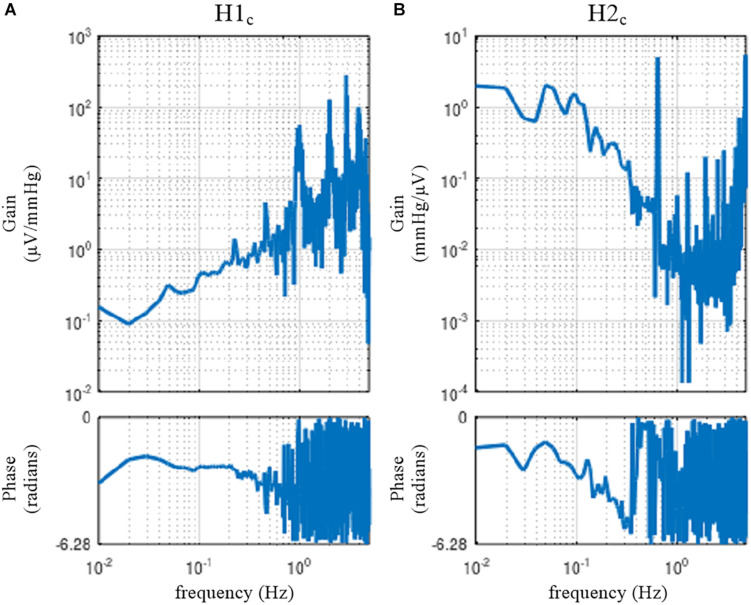
The neural arc **(A)** and peripheral arc **(B)** transfer functions as estimated by a closed-loop identification method with an exogenous pressure perturbation by blood infusion and withdrawal. H1_*c*_ and H2_*c*_ were derived from Eqs. 4.2.3 and 4.2.6, respectively.

Figure 15 illustrates the results of a closed-loop identification, obtained from the following codes:

A = recread(′c:/SampleData\rabbit4-closed.dat′, 4);

B = recresample(A, 20); figure, recplot(B, 10);

[H1c H2c] = opeclo(B, [1 3 2]);

figure, subplot(1, 2, 1), tfplot(H1c, ′r′); subplot(1, 2, 2), tfplot(H2c, ′r′);

The user-defined function **opeclo** calculates the open-loop transfer functions from the closed-loop data with an exogenous pressure perturbation. The second argument of **opeclo** specifies the channels in the following order: the command signal, the AP signal that is directly affected by the exogenous perturbation, and the SNA signal. In Figure 15, the estimated H1_*c*_ and H2_*c*_ captured the derivative and low-pass characteristics, respectively. The phase plot of H1_*c*_ revealed the out-of-phase relationship with a slightly convex-upward shape. The phase plot of H2_*c*_ showed that the phase approached 0 radians at the lowest frequency and delayed with increasing frequency. In contrast to Figures 11, 12, the phases of H1_*c*_ and H2_*c*_ did not show a fixed relation of angle(H1_*c*_) = −angle(H2_*c*_). However, the estimation of H1_*c*_ in the frequency range above 0.5 Hz was more dispersed than that derived from the open-loop experiment (Figure 3). This is partly because the switching interval for the blood infusion and withdrawal was 1 s, and the input power of the exogenous pressure perturbation decreased in the frequency range above 0.5 Hz. The estimation of H2_*c*_ in the lower frequency range was more dispersed than that derived from the open-loop experiment (Figure 4). This result is partly because the estimation of H2_*c*_ relies on the unknown endogenous noise component of SNA. There were no statistically significant differences between the transfer function parameters estimated via the closed-loop method and those estimated via the open-loop method in the same animals, excepting *f*_*c*__2_ in the neural arc (Kawada et al., 1997). The parameter *f*_*c*__2_ was not compared because the lack of input power in the higher frequency range hampered a reliable estimation of *f*_*c*__2_ in the closed-loop method.

## Limitations

First, we did not treat the cardiac branch of the arterial baroreflex partly because most of the data were obtained from anesthetized animals with vagotomy. The vagal branch exerts a rapid HR response compared with the sympathetic branch (Kawada et al., 2019). For the analysis of clinical data, whether the dynamic properties of the cardiac branch can be estimated accurately under conditions with the intact vagi needs to be examined because cardiac vagal nerve activity predominantly regulates HR in humans under resting conditions (Hori and Okamoto, 2012).

Second, the AP regulation involves mechanisms other than the arterial baroreflex such as autoregulation and blood volume control. The autoregulation manifests in association with blood flow control. For instance, cerebral blood flow is autoregulated near constant over a wide pressure range, while it could be more pressure passive during low cardiac output conditions (Lie et al., 2021). The blood volume control by the kidneys is essential for long-term AP control. An open-loop analysis on the baroreflex-mediated AP changes and associated urine output function may provide a clue to connect the arterial baroreflex and the blood volume control (Kawada et al., 2020). Further studies are clearly required for an integrative understanding of the AP regulation.

## Conclusion

Although there are many closed-loop identification studies, validation is made indirectly on the basis of the predictability of the output signal from the input signal. In this article, we compared the results of a closed-loop identification with the open-loop dynamic characteristics of the baroreflex system. Although the frequency-domain closed-loop identification employing an exogenous pressure perturbation was successful in separately assessing the transfer functions of the neural and peripheral arcs, there remains an issue of estimation accuracy in the higher frequency range of the neural arc and in the lower frequency range of the peripheral arc. Further efforts are required to identify open-loop dynamic characteristics of the arterial baroreflex system from closed-loop data. Alternatively, a priori knowledge about the open-loop dynamic characteristics of the arterial baroreflex system may be used to advance the assessment of baroreflex function under closed-loop conditions. In this regard, Mannoji et al. (2019) proposed that the ratio of power spectra of AP between two frequencies be used to derive an index of baroreflex gain. Clinical data demonstrated that aging steepens the slope of the AP power spectra, probably reflecting the deterioration of the arterial baroreflex in older subjects (Mano et al., 2021). The noninvasive nature of the AP power spectral analysis is an advantage of the method. On the other hand, information on the index of baroreflex gain alone is insufficient to construct a cardiovascular simulator that enables the prediction of dynamic AP changes in response to interventions. Clinical application of closed-loop identification of baroreflex properties awaits further investigations.

## Data Availability Statement

The original contributions presented in the study are included in the article/supplementary material, further inquiries can be directed to the corresponding author/s.

## Ethics Statement

The animal study was reviewed and approved by The Animal Subject Committee at the National Cerebral and Cardiovascular Center.

## Author Contributions

TK prepared the figures and drafted the manuscript. All authors discussed the contents of the manuscript, edited and revised the manuscript, and read and approved the final manuscript.

## Conflict of Interest

The authors declare that the research was conducted in the absence of any commercial or financial relationships that could be construed as a potential conflict of interest.

## Publisher’s Note

All claims expressed in this article are solely those of the authors and do not necessarily represent those of their affiliated organizations, or those of the publisher, the editors and the reviewers. Any product that may be evaluated in this article, or claim that may be made by its manufacturer, is not guaranteed or endorsed by the publisher.

## Appendix

### Setup of GNU Octave

GNU Octave is available at https://www.gnu.org/software/octave/. The sample codes were tested using GNU Octave, version 6.2.0 (Local) (GUI). After installing the GNU Octave, the path for user function files (*.m) needs to be added through the [SetPath] command in the [Edit] menu. Execute “demo_open” to generate Figures 1–8. Execute “demo_closed” to generate Figures 11–15.

### Setup of Scilab

Scilab is available at https://www.scilab.org. The sample codes were tested using Scilab version 6.1.0 (64-bit) Desktop. Open “dynamic_step.zcos” in Xcos after initializing the parameters used in the block diagram (see section “Design of a Block Diagram”).
